# Experimental Study of Sinkhole Propagation Induced by a Leaking Pipe Using Fibre Bragg Grating Sensors

**DOI:** 10.3390/s24196215

**Published:** 2024-09-25

**Authors:** Josué Yumba, Maria Ferentinou, Michael Grobler

**Affiliations:** 1 Department of Civil Engineering Science, University of Johannesburg, Johannesburg P.O. Box 524, South Africa; 2 School of Civil Engineering and Built Environment, Liverpool John Moores University, Liverpool L33AF, UK; m.ferentinou@ljmu.ac.uk; 3 Department of Electrical and Electronic Engineering Science, University of Johannesburg, Johannesburg P.O. Box 524, South Africa; michaelg@uj.ac.za

**Keywords:** sinkhole modelling, fibre Bragg grating, FBGs, optical fibre sensors

## Abstract

Sinkhole formation caused by leaking pipes in karst soluble rocks is a significant concern, leading to infrastructure damage and safety risks. In this paper, an experiment was conducted to investigate sinkhole formation in dense sand induced by a leaking pipe. Fibre Bragg grating (FBG) sensors were used to record the strain. A balloon was gradually deflated within a bed of wet silica sand to create an underground cavity. Eighteen FBG sensors, with a wavelength range between 1550 nm and 1560 nm, were embedded horizontally and vertically in the physical model at different levels to monitor deformation at various locations. A leaking pipe was installed to induce the collapse of the formed arch above the cavity. The strain measurements suggested the following four phases in the sinkhole formation process: (1) cavity formation, (2) progressive weathering and erosion, (3) catastrophic collapse, and (4) subsequent equilibrium conditions. The results showed differences in the strain signatures and distributions between the horizontal and vertical measurements. During the critical phase of the sinkhole collapse, the horizontal measurements primarily showed tension, while the vertical measurements indicated compression. This investigation demonstrates the effectiveness of FBGs as advanced monitoring tools for sinkhole precursor identification. The study also suggests using FBGs in geotechnical monitoring applications to improve the understanding and mitigation of sinkholes and related geohazards.

## 1. Introduction

Sinkholes and land subsidence are dangerous natural (or human-induced) hazards resulting in fatalities and property loss, often affecting transportation and other infrastructure networks in regions under soluble bedrock. In recent decades, an increase in sinkhole-related damage has been reported globally, and this has been attributed to human activities that initiate mobilising agents or to the development of sinkhole-prone terrain [1,2,3].

In urban environments, sinkholes and subsidence pose a significant risk to the affected communities and infrastructure [4,5,6,7]. The examination of recent instances was drawn from various countries and locations, including the USA, Florida [8] and Virginia [9]; Italy [10,11,12]; Turkey [13]; China [14]; Saudi Arabia [15]; the Dead Sea [16], and South Africa [17,18,19].

These incidents frequently manifest suddenly with minimal or no advance notice and can cause devastating impacts without readily detectable forewarnings. The primary financial burden is linked to the remediation, mitigation, and closure of the revealed cavity, followed by the secondary costs of repairing the impacted infrastructure or property. Sinkhole-related losses can be classified as either direct or indirect. Direct losses relate to fatalities and damage to property and infrastructure (for example, damage to service lines, roads and pipelines, and differential settlements in building support foundations). In contrast, indirect losses extend on a larger scale and can impact entire communities or businesses, reaching beyond the immediate vicinity. This includes disturbances to business operations, transportation, and communication networks. The associated costs encompass assistance, temporary storage, and accommodation for individuals displaced by the event. Additionally, intangible losses are challenging to quantify monetarily, such as disruptions in daily commuting, mental health impacts, and, ultimately, the expenses tied to relocating the population from high-risk areas [12].

Between 2000 and 2015 in the USA, the average cost of sinkhole-associated damage was estimated to be at least US $300 million per year, with the total likely much higher [20]. Located on expansive gypsum formations, the Spanish cities of Oviedo (1998) and Calatayud (2003) experienced significant economic setbacks due to individual collapse incidents, amounting to 18 million and 4.8 million euros, respectively [21]. Furthermore, around 30,000 households were moved to a more secure location on dolomite terrain west of Johannesburg, incurring a substantial expense exceeding US $600 million [1].

Sinkholes can be classified into the following three main categories based on the processes leading to their formation [4]: collapse sinkholes, characterised by the brittle gravitational deformation of cover, bedrock, or caprock material; sagging sinkholes, where ductile sediments sag due to the differential lowering of the cover deposit, bedrock, or caprock; and suffusion sinkholes, involving the migration of cover deposits downward through dissolution conduits accompanied by ductile settling. In practical terms, many sinkholes emerge due to a combination of these processes, such as the following: solution sinkholes, collapse sinkholes, caprock sinkholes, dropout sinkholes, suffusion sinkholes, and buried sinkholes [4].

The mechanism behind the formation of sinkholes involves the ongoing dissolution of subsurface soluble rocks, gradually giving rise to voids. As the soil above these voids reaches a critical state, marked by the surpassing of shear strength by soil material, it undergoes collapse due to diminished vertical soil stress. Primary contributors to underground void formation include water infiltration, groundwater table reduction, or extensive pumping resulting in dewatering [12,17].

Drawing insights from the South African dolomites, Jennings et al. [22] elucidated the geotechnical failure mechanism of sinkholes, attributing it to the collapse of an arch or dome spanning an air-filled void. The collapse is characterised by an “onionskin” peeling of the intrados, causing the material to fall into the void, subsequently moving the void toward the ground surface. The culmination of this process results in the manifestation of a sinkhole. Jennings outlines five requirements for sinkhole formation as follows:

1. Sturdy material supports the soil covering the dome, functioning as essential support for the void’s roof. The span should fall within the limits conducive to arch formation.
2. The development of an arching mechanism within the residuum material.
3. The formation of a void beneath the arch in the residuum.
4. The requirement for a reservoir below the arch to accumulate the material resulting from the arch’s collapse.
5. The initiation of a disruptive factor occurs when a void of adequate size forms in the residuum, leading to the collapse of the arch’s roof. Typically, the triggering factor is water infiltration, causing a weakening of the arching soils.

Water infiltration into the soil serves as a trigger, increasing the load of soil material deposited in the vadose zone. Consequently, this has the potential to diminish the geomechanical strength of the soil and facilitate internal erosion and dissolution processes [21].

The present study investigates the propagation of a sinkhole caused by a leaking pipe in dense sand. It analyses the significance and methods used in sinkhole monitoring, introduces optical fibre sensing with fibre Bragg gratings (FBGs), describes the experimental setup and data collection approach, presents and discusses the findings, and concludes on the effectiveness and potential applications of FBG sensors in geotechnical monitoring and mitigation.

## 2. Sinkhole Monitoring Methods and Previous Investigations

### 2.1. Methods of Sinkhole Monitoring

The techniques and technologies commonly employed for monitoring sinkhole deformations have undergone extensive testing and application in other geohazard monitoring domains. However, when dealing with cavity propagation, there are inherent challenges compared to landslide hazards [12]. One challenge is the sudden manifestation of the phenomenon without any warning, particularly in the case of sinkhole caprock collapse [4]. According to Intrieri et al. [12], measurable plastic deformation may occur days before the failure, ranging from a few millimetres to a few centimetres. Observations from numerical simulations and physical model experiments by [23,24,25] suggest that while surface subsidence accelerates during the precursory period, the width of the subsiding area remains unchanged. This indicates that the upward propagation and breakthrough of the cavity do not necessarily occur during the precursory period.

Over the past few decades, various detection and monitoring methods have been extensively employed to investigate sinkholes and subsidence. These approaches include GPS and geophysical techniques such as electrical resistivity tomography (ERT), ground-penetrating radar (GPR), cross-hole electrical resistivity tomography (CHERT), and seismic refraction (SR) [6]. Another widely used method is the Interferometric Synthetic Aperture Radar (InSAR), which utilises satellite radar images to create interferograms that are analysed to detect changes in the earth’s surface. Optical fibre sensing technology has recently been proposed for detecting and monitoring sinkhole formation [3,24,25,26].

In many sinkhole locations, gradual land subsidence persists for weeks to years following the collapse. Therefore, a quantitative assessment of subsidence rates and their spatial and temporal extents provides valuable insights into subsurface sinkhole formation processes. It enables the evaluation of activity levels at specific sites and distinguishes between active and inactive areas.

### 2.2. Previous Investigations of Sinkholes

The available literature shows that various researchers have investigated soil behaviour and sinkhole formation through analytical, physical, and numerical modelling. Terzaghi [27] investigated stress distribution in the sand over a yielding trap door and found that stress was transferred away from the trap door under pressure. Costa et al. [28] examined failure mechanisms in granular soil simulating deep and shallow conditions over a deep trap door. Among their findings was that the relative density influenced the surficial settlement. Alrowaimi et al. [29] used physical models to show a groundwater cone of depression to indicate imminent sinkhole collapse. Jacobsz [23] found that the zone of influence of a trap door matched its width, indicating that sinkhole diameters are like the arch width. Al-Naddaf et al. [30] demonstrated that geosynthetics stabilised soil arching and increased load capacity under surface loading. Labuschagne et al. [24] and Ferentinou [25] used fibre Bragg grating sensors to monitor sinkhole propagation, with their findings highlighting these sensors’ sensitivity to the sand relative density’s effect on failure patterns. Möller et al. [26] showed that distributed fibre optic sensing allowed for sinkhole prediction formation, suggesting its potential for early warning systems in critical infrastructure.

The literature on sinkhole monitoring has garnered increasing interest due to the growing need to understand the mechanisms of incipient collapse and monitor ground displacement rates for effective risk management. Reliable spatial and temporal hazard mapping, supported by monitoring programs, can enhance sinkhole risk management by adopting sinkhole protocols, land zoning, remedial measure design, and performance assessment of these solutions. Analysing research trends highlights the need for future studies to advance sensor technologies, enhance the understanding of sinkhole and soil behaviour, integrate physical models, examine environmental impacts, and apply these findings to critical infrastructure to improve early detection and mitigation of sinkhole risks.

## 3. Introduction to the Optical Fibre Sensing Using Fibre Bragg Gratings (FBGs)

Optical fibre sensing (OFS) technology employs optical fibres as sensors to measure diverse physical quantities like strain, temperature, pressure, and vibration. Optical fibre sensors present several advantages compared to conventional sensing technologies, including high sensitivity, immunity to electromagnetic interference, and the capacity to cover extensive distances without signal degradation. OFS utilises dielectric devices that confine and guide light [31]. The optical fibre comprises a glass core surrounded by a glass cladding with a refractive index lower than the core, facilitating the propagation of light waves within the core. The buffer coating safeguards the glass fibre, and, at times, an outer jacket provides mechanical protection to the fibre.

**Figure 1:**
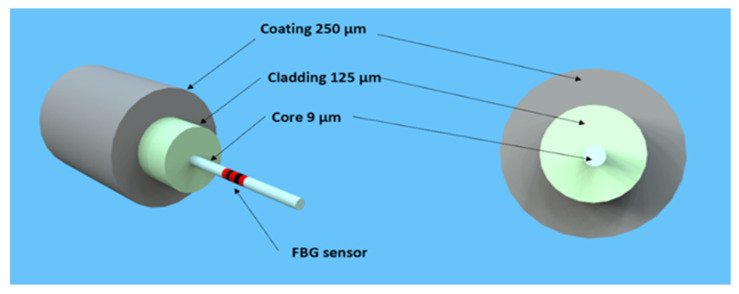
Depiction of a single-mode optical fibre.

Fibre Bragg gratings (FBGs) represent a category of optical fibre sensors widely applied in geotechnical and structural monitoring applications. FBGs consist of an optical fibre section with a periodically changing refractive index. These periodic variations result in a wavelength-specific reflection of incident light known as the Bragg wavelength. Figure 1 illustrates a single-mode fibre with a printed FBG sensor.

An FBG sensor monitoring system can determine strain or temperature change based on the wavelength change in the reflected light signal at the grating position [32,33]. The reflected Bragg wavelength is shown in Equation (1) as follows:

(1) $$\lambda_{B}=2n_{eff}\Lambda$$

In the given expression, where *λ_B_* represents the Bragg wavelength, $n_{eff}$ denotes the effective refractive index of the optical fibre core, and Λ represents the grating period. When strain is applied to the sensor, causing a change in the grating period (Λ), there is a corresponding shift in the reflected Bragg wavelength, as indicated in Equation (1). The fundamental detection principle of the FBG sensor is expressed by Equation (2) as follows:

(2) $$\frac{\Delta \lambda_{B}}{\lambda_{B}}=\left(1-p_{e}\right)\varepsilon +\left(\alpha_{\Lambda }+a_{n}\right)\Delta T$$

In the given expression, ∆*λ_B_* is the change in the reflected wavelength, *λ_B_* denotes the initial Bragg wavelength, *p_e_* stands for the photoelastic coefficient, ε represents the strain, $\alpha_{\Lambda }$ is the thermal coefficient, $a_{n}$ is the thermal modulation of the core refractive index, and ΔT is the temperature change [33].

If we replace $\left(1-p_{e}\right)$ by β and $\left(\alpha_{\Lambda }+a_{n}\right)$ by ξ, Equation (2) can be written in a simplified form as Equation (3) as follows:

(3) $$\frac{\Delta \lambda_{B}}{\lambda_{B}}=\beta \varepsilon +\xi \Delta T$$

The coefficients related to strain and temperature β and ξ can be found by calibration.

**Figure 2:**
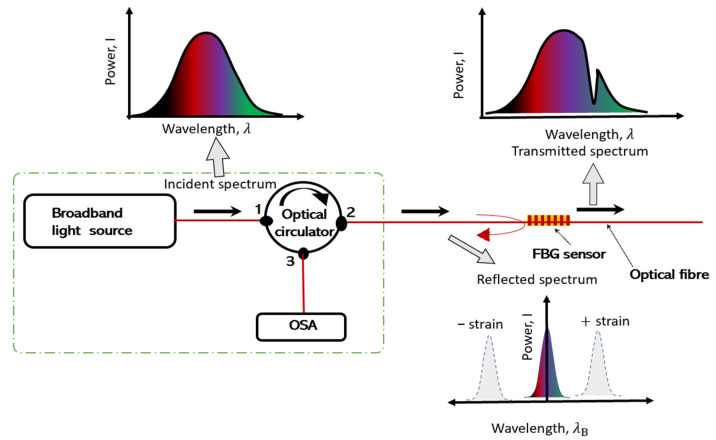
Working principle of an FBG sensor.

When the FBG sensor is stretched or compressed, the reflected Bragg wavelength of the FBG varies. It is, nevertheless, also sensitive to changes in temperature. As a result, strain and temperature fluctuations affect the FBG. Figure 2 illustrates the pathway of an incident spectrum from the broadband source as it travels through the optical fibre with an FBG sensor, and the reflected spectrum subsequently being [32].

## 4. Materials and Methods

Physical modelling in geotechnical engineering is a physical representation of a field prototype in an investigation. The scaling law applied in this study was designed to ensure similarity between the field prototype and the physical laboratory model by adhering to geometric and dynamic similarity principles. The scaling approach can influence the interpretation of results, particularly affecting the distribution of stress and strain observed in the model. While the laboratory model offers valuable insights, it is crucial to consider the impact of scaling when extrapolating these results to real-world scenarios, especially regarding deformation and collapse dynamics. Several challenges can arise in a 1-g model, such as differences in stress paths, difficulties in replicating compaction effects, and boundary effects like friction and adhesion, which can cause discrepancies in accurately simulating full-scale prototype behaviour, leading to non-uniform stress distributions and localised strains [34].

Similitude laws can be derived using the π-theorem for dimensional analysis [35]. However, reduced-scale models often have lower stress levels than full-scale structures, leading to different soil properties and loading conditions [34]. Additionally, physical models cannot fully meet similitude requirements, and tests at a low relative density at 1-g may correspond to a higher relative density in the field [35].

The parameters for 1-g proposed by [36] were utilised to scale down the model, as shown in Table 1. A specific scale ratio (N = 1:30) replicates the geometric proportions of the sinkhole and surrounding soil. A field site measuring 15 × 8.1 × 2.4 m was scaled down using a factor of 30, enabling the construction of an experimental box model with dimensions of 500 × 350 × 80 mm; the soil was filled and compacted to a height of 270 mm, representing 8.1 m in the field prototype. This scaling factor was selected, consistent with previous sinkhole models developed in the same laboratory by Labuschagne et al. [24] and Ferentinou [25]. This approach allowed for the study of stress and strain distribution, deformation, and collapse dynamics in a controlled environment.

Fine silica sand was used in this experiment, which was obtained from the Cullinan mine, a commercial source near Pretoria in the Gauteng province, South Africa. The properties of silica sand are described in Table 2. Since the compaction test found the soil’s optimal moisture content (OMC) was 15.6%, it was mixed at 10% moisture content to avoid being too wet or saturated. This 10% moisture level was sufficient to create a suction effect in the non-cohesive soil, allowing a cavity to form and providing suitable conditions to study the impact of infiltration of leaking water over an extended period.

The soil was placed in layers and compacted to a thickness of 30 mm, achieving a density of 1548.8 kg/m³ and a relative density of 60.7%, classifying it as dense. The arch interface formed between the balloon and the sand was determined based on the relative density of the compaction in the model. Compared to models developed earlier in the same laboratory [27,28], the soil used was considered in wet conditions to simulate the most encountered conditions in the field.

Additionally, in this experiment, a leaking pipe caused a collapse. This collapse resulted from water-reducing soil cohesion, known as suction or apparent cohesion. Also, the position of the sensors was combined vertically and horizontally, while in previous works, only the horizontal placement of sensors and dry silica sand.

The phase mask technique was used to print the FBG sensors for this study in the University of Johannesburg Photonics Laboratory. The fourth harmonic (266 nm) of a Nd/YAG laser was used to print the gratings in the wavelength range of 1550 nm to 1560 nm. The FBGs were calibrated to determine the strain and temperature coefficients (*β* and *ξ*) as stated in Equation (3). The coefficients *β* and *ξ* of the FBG sensors were found to be 7.06 × 10^−7^ and 7.14 × 10^−6^/°C.

The FBGs were printed on seven (7) different optical fibre cables. Each optical fibre had three printed sensors with a 60 mm interval between the sensors, corresponding to a 1.8 m interval in the field prototype. One fibre was used to measure temperature to allow for temperature compensation. Three fibres were placed in horizontal positions, and three were placed in vertical positions above the cavity to measure strain. Eighteen FBG strain sensors were placed to capture signatures of the strain field in the expected damage zone.

**Figure 3:**
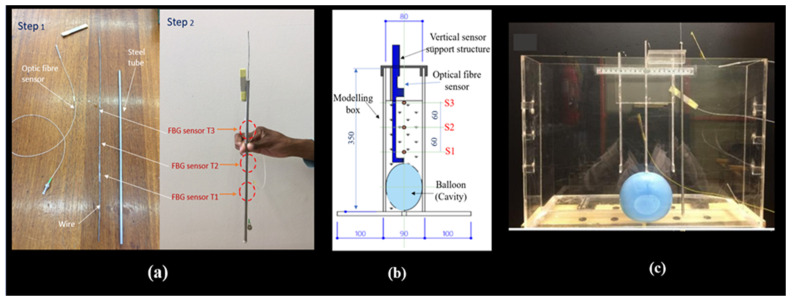
Model preparation: (**a**) Isolation of temperature cable with three printed FBGs and T1, T2, T3 in red show the locations of temperature sensors isolation tube, (**b**) vertical support design of the optical fibre sensors mounted in the modelling box with S1, S2, S3 in red show the locations of the strain sensors, and (**c**) container with optical fibre sensors mounted.

The optical fibre temperature was encased in an aluminium tube to isolate temperature sensors from strain or vibration disturbances during the experiment. This setup also allowed for temperature compensation in determining strain values measured by strain sensors, as shown in Figure 3a. Figure 3b illustrates a cross-section of the vertical support structure to which the optical fibre cables were attached and maintained vertically. The cables were fixed at both ends to the support structure using superglue, and their bodies were coated with silicone and rolled in sand to create an adhesive surface, ensuring strong adherence to the sand.

The container (experimental box) was constructed from Perspex material (Figure 3c), enabling the complete burial of the optical fibre sensors in the soil. This design ensured efficient soil deformation detection without compromising measurement accuracy due to structural influence.

**Figure 4:**
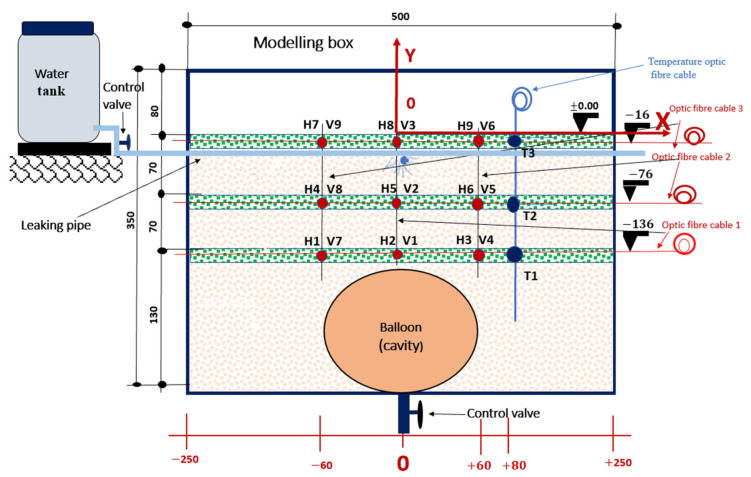
Horizontal and vertical positions of FBG sensors in the sinkhole model. The green-dotted layers represent the dyed soil, visually illustrating the collapse pattern. ’H’ with a number indicates the horizontal positioning of a specific FBG sensor, while ’V’ with a number denotes the vertical placement of the sensor.

**Figure 5:**
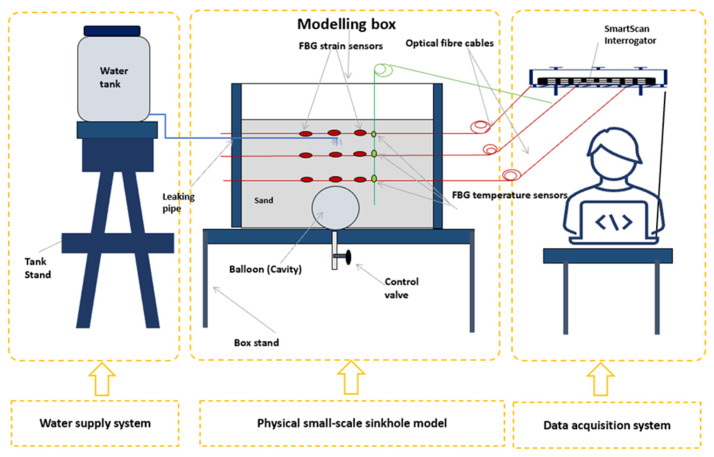
Experimental setup consisting of water supply system, physical small-scale model of the sinkhole, and data acquisition system.

The experimental setup consisted of the following three main parts: the water supply system, the container, the data acquisition system, and the instrumentation, as illustrated in Figure 4 and Figure 5. The water tank served as a source for the water. A control valve was mounted at the bottom of the tank to regulate the flow rate. The fibre optic sensors were placed at specific locations in the model to monitor deformation, as shown in Figure 4. In the vertical direction, optical fibre sensors were placed 16 mm below the surface in the model, corresponding to a depth of 0.5 m in the field prototype; the second set of optical fibre sensors was placed at a depth of 76 mm, corresponding to a depth of 2.3 m in the field prototype; and finally, the third set of optical fibre sensors was placed at a depth of 136 mm, corresponding to a depth of 4.1 m in the field prototype. The balloon was positioned at a depth of 150 mm, equivalent to 4.5 m in the field, with a diameter of 120 mm, corresponding to 3.6 m.

A water-filled balloon was used to create a cavity at the bottom of the model. The balloon was inflated to a volume of 550 mL, with a horizontal diameter of 115 mm. The balloon’s volume and diameter defined the dimensional characteristics of the cavity formed in the soil mass after compaction, corresponding to the void of a volume of 14.85 m^3^ and a diameter of 3.45 m in the field. A sinkhole that can form with this diameter of two to five metres can be classified as medium size [37]. The experiment was performed in a controlled environment. The water used in the experiment to mix soil and supply the leaking pipe was kept in the same conditions (experimental room) for 24 h with the silica sand to minimise temperature fluctuations and prevent thermal expansion or contraction effects.

A leaky pipe was placed 30 mm below the soil’s surface according to the South African specifications of trench requirements for flexible pipes [38]. The cavity formation period was the period it took to deflate the balloon at a flow rate of 55 mL/m. After the cavity was formed, water was leaked into the model through the buried leaky pipe at a flow rate of 40 mL per minute, calibrated for inducing the collapse of the cavity arch in a control condition and allowing the proper collection and analysis of the data during this period, while the FBGs were interrogated with a SmartScan interrogator.

## 5. Results

**Figure 6:**
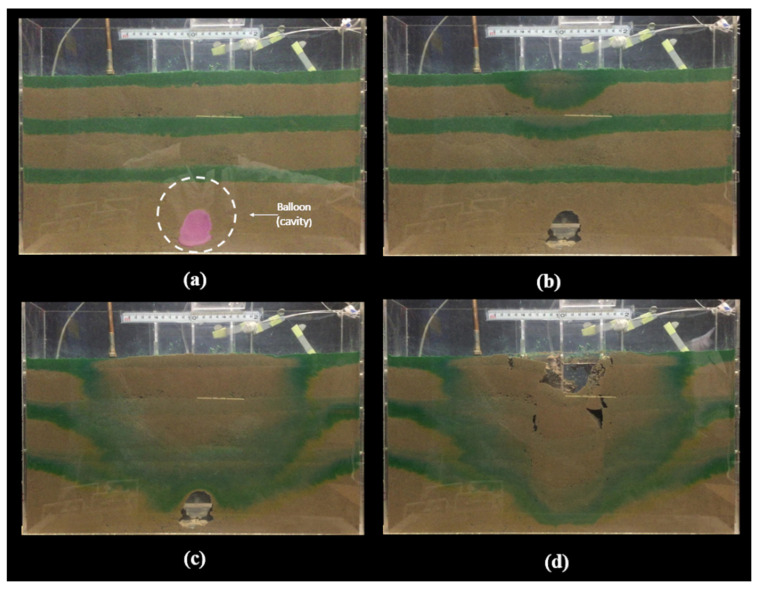
Development of the sinkhole: (**a**) The balloon is inflated with water to create a cavity in the soil mass. (**b**) The ballon is deflated, and the cavity is created. (**c**) Water leaks into the soil mass to induce the collapse and form the sinkhole. (**d**) The sinkhole develops and is propagated upward toward the ground surface.

Figure 6 depicts the progression of a sinkhole, starting from cavity formation, followed by the weathering and erosion of the soil mass, and culminating in the collapse of the cavity and its upward propagation. The dyed soil layer indicates water infiltration from a leaking pipe, which triggers the cavity’s collapse. In Figure 6a, a balloon is inflated with water to create a cavity in the soil mass. Figure 6b shows the balloon’s deflation and the cavity’s formation. In Figure 6c, water leaks into the soil mass, causing the cavity to collapse and form a sinkhole. Finally, Figure 6d shows the sinkhole’s development and upward propagation to the ground surface.

**Figure 7:**
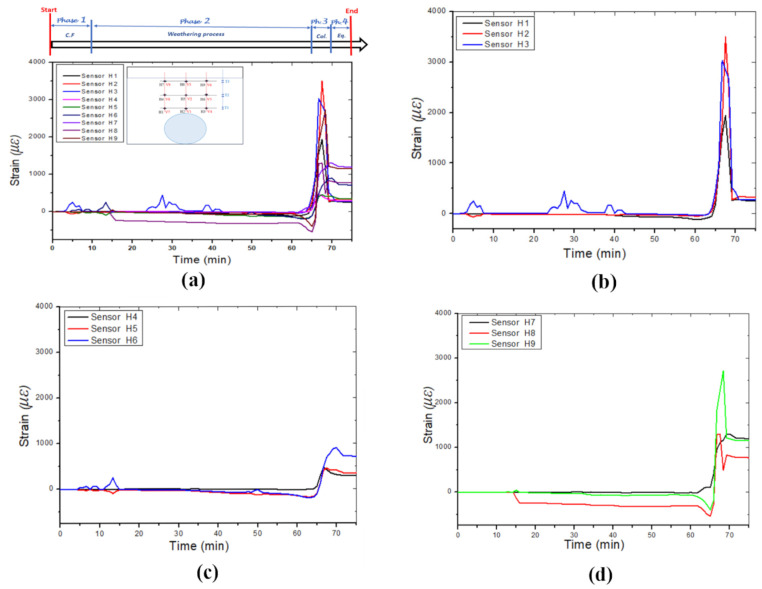
The horizontal strain was measured in different layers by three optic fibre sensor cables: (**a**) Strain induced by leaking water, (**b**) optic fibre cable 1, (**c**) optic fibre cable 2, and (**d**) optic fibre cable 3.

Figure 7a illustrates the horizontal strain signatures measured by the horizontally positioned FBG sensors and highlights the boundaries of the four identified phases. The overall trend of the strain signatures in this model indicates that the FBG sensors experienced tensile deformation during the collapse period. FBGs H1, H2, and H3, placed above and closer to the cavity, recorded the highest strains due to their proximity (Figure 7b). FBG H2 measured the maximum strain of approximately 3.5 × 10³ με. FBGs H4, H5, and H6 recorded lower strain values (Figure 7c). During the weathering period, FBGs H5, H6, H8, and H9 displayed strain variations before the collapse, likely due to the leak orientation affecting the right-hand side of the model more than the left.

**Figure 8:**
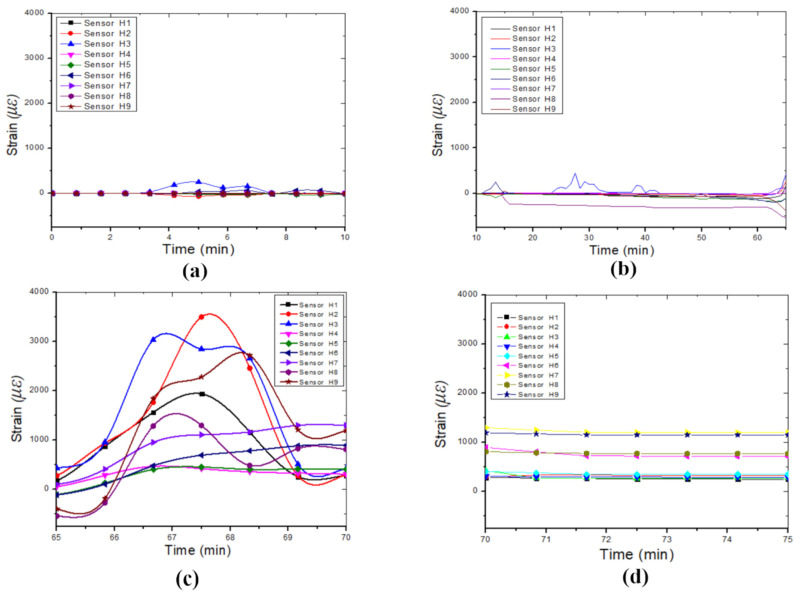
Horizontal strain variation during the four identified phases in the failure process of the sinkhole: (**a**) Cavity formation process, (**b**) weathering or erosion process, (**c**) collapsing process, and (**d**) equilibrium process.

Figure 8 illustrates the strain variation observed in the four identified phases of the failure collapse process. Figure 8a shows the strain variation during cavity formation, i.e., while the balloon deflates. Figure 8b displays the strain variation while water was leaked into the soil mass (during the weathering and erosion process); most of the FBGs were in a small compression state before the failure occurred. The strain variation measured during the collapse of the cavity is displayed in Figure 8c, and the post-collapse phase is represented in Figure 8d, where the variation in the strain is relatively constant.

**Figure 9:**
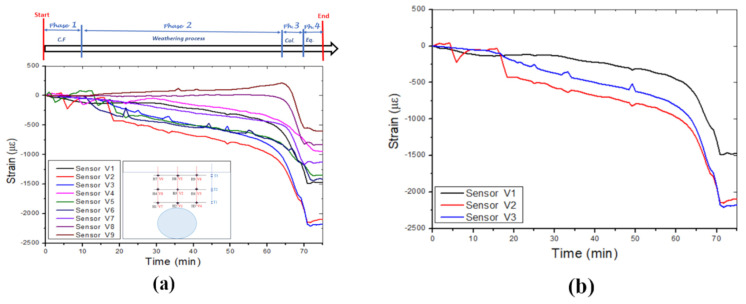

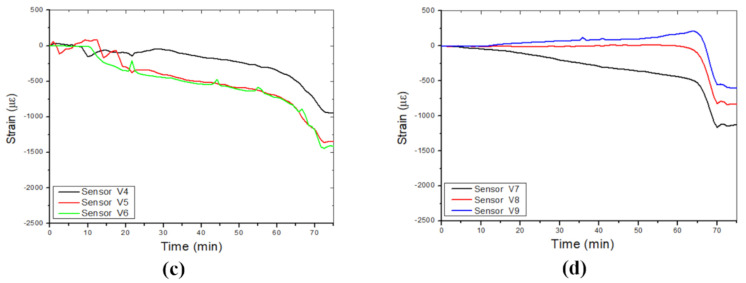
Vertical strains were measured in different layers by the three optic fibre sensor cables: (**a**) Strain induced by leaking water, (**b**) optic fibre cable 1, (**c**) optic fibre cable 2, and (**d**) optic fibre cable 3.

Figure 9a displays the strain variations measured by all the vertically positioned FBG sensors and the boundaries of the four identified phases. The strain level is lower than those measured by the horizontally positioned sensors, except for FBGs V2 and V3. FBGs V2 and V3 measured the highest strain variation (maximum strain, approximately 2.25 × 103 με) on cable one (1), owing to its location in the central axis above the cavity, as shown in Figure 9b.

The strain variation on the right side of the cavity is presented in Figure 9c, and FBG V6, located in the middle, measured the maximum strain value at approximately 1.5 × 10^3^ με. However, the strain variation on the left side of the cavity is represented in Figure 9d, where V7 measured a maximum strain of approximately 1.15 × 10^3^ με at the cavity roof. Moreover, the cable on the left side of the cavity detected lower strain values. The vertical strain variation due to the leaking water that induced the collapse of the cavity is also shown in Figure 9d.

**Figure 10:**
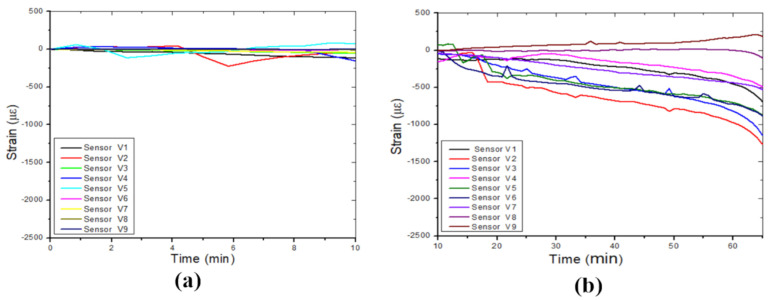

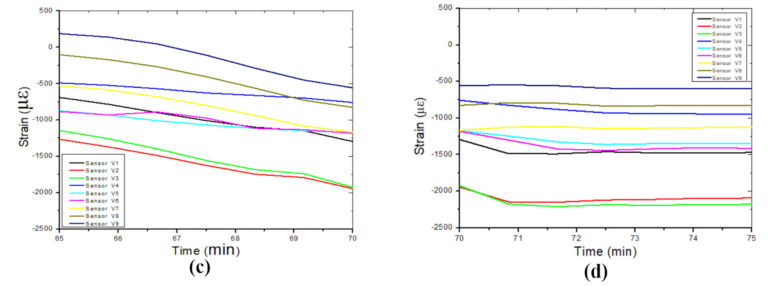
Vertical strain variation during the four identified phases in the failure process of a sinkhole, (**a**) cavity formation process, (**b**) weathering or erosion process, (**c**) collapsing process, and (**d**) equilibrium process.

Figure 10 shows the strain variation during the four identified phases of the failure process. Figure 10a shows the strain variation during the cavity formation (while the balloon was deflating), and Figure 10b displays the strain variation while water leaked into the soil mass (during the weathering and erosion process). During this period, the FBGs experienced a gradual compressive deformation until failure occurrence. The strain variation measured during the cavity collapse is displayed in Figure 10c, and the post-collapse phase is illustrated in Figure 10d, where the strain variation is relatively constant.

## 6. Discussion

The reduced-scale physical model developed to investigate the sinkhole propagation in partially saturated dense sand corresponds closely to the reality of the field prototype since, in most cases, the subsoil is in a wet or partially saturated state. Five conditions must be met for a sinkhole to form, as described by [22]. These conditions were considered during the experimental design process.

Installing optical fibre sensor cables at various depths aimed to identify strain distribution, revealing that sensors near the cavity recorded significantly higher strains than those at the surface or middle. The study, focused on shallow sinkholes, may not reflect deeper conditions where stress effects are more expected.

The results reveal that the formation and the upward propagation of the sinkhole pass through the following four phases: an underground cavity formation, a weathering process accompanied by an erosion process, a collapsing process, and an equilibrium process. The time taken to deflate the balloon (10 min) determined the underground cavity formation period. The cavity was formed according to the volume/diameter of the inflated balloon. The FBGs detected the movement of the induced internal deformation of the soil mass when the arch formed. Usually, a trigger would weaken the arch until it collapsed [39].

Weathering is the deterioration of rock by chemical, physical, or biological processes [40]. In this experiment, this period was characterised by the erosion time (55 min) just after the cavity formed. The water leaked into the soil mass during the period when water was leaked from the pipe. This overburdened soil sank into the cavity, changing the bonding strength between the soil grains and decreasing the mass of the soil at the surface [39]. This process continued upward in the shape of an arch until the soil remaining in the arch eventually collapsed and sank into the cavity [39]. The flow rate was 40 mL/min, equivalent to approximately 7.27% of the original cavity volume per minute. The leaking pipe leaked water into the soil for 55 min. The total amount of water that induced collapse was 2200 mL, compared to the original cavity volume of 550 mL, representing a ratio of 1:4.

Both horizontally and vertically positioned FBG sensors experienced compressive deformation during the leaking period, particularly the vertically positioned sensors. During the collapse period, the strains measured by the FBGs increased significantly to a maximum value. The vertical sensors experienced compressive deformation, while the horizontal sensors experienced tension deformation during the collapse of the cavity. These differences in strain signatures are crucial to identifying sinkhole formation phases; the horizontal tension indicated lateral spreading, while the vertical compression suggested settlement. Analysing these signatures over time allows for the tracking of the critical phases of sinkhole development, from the initial cavity formation to soil erosion caused by the leaking water and the critical collapse. This analysis validated the effectiveness of FBG sensors in detecting various strain modes.

Comparing the findings with [26] in the 1-g experiment, the vertical strain profile showed no negative strains, while the horizontal profile did, with larger magnitudes in the horizontal direction. The FBG sensors were in a tensile state, indicating tensile deformation primarily occurred horizontally. In contrast, [41] found that vertical fibres at 10-g experienced compressive states during the centrifuge test, suggesting soil compression with generally small compressive strains. Before the collapse, most horizontal FBG sensors recorded negative strains or compression before transitioning to a sudden tensile peak of the collapse, as observed by [3], where the ground deformation in stage 2 caused a gradual shift from compressive to tensile strains.

FBG sensor readings are influenced by their position, orientation, setup, and soil moisture. Compared to the previous balloon deflation experiments in dry sand [27,28], this study used wet sand, altering the failure mechanism. A cavity was formed by suction in the wet sand, with water infiltration weakening the arch and causing collapse. The horizontal sensors showed consistent tensile strain patterns, aligning with their results [27,28].

The finding revealed that the internal deformation mechanism’s distribution was not symmetrical, implying that the strain distribution is not similar on both sides (the left and right sides of the cavity). This could be due to the orientation of the leak point in the soil mass, the infiltration of the water, and the compaction mode, as this was affected by the arrangement of the sensors and their different support devices in the model.

In the last phase, identified as the equilibrium process, the graphs showed no strain variations for all the sensors. The soil reached a balanced state.

However, the horizontal installation of optical fibre cables at significant depths in urban environments can pose considerable challenges. Conversely, vertical installations can offer advantages by simply requiring a small borehole to insert the sensing cable attached to its anchor or vertical support system (smart anchoring system). When properly calibrated and deployed, FBG sensors are expected to perform reliably even in less controlled real-world environments. Although soil heterogeneity and unpredictable weather may introduce variability, the sensors’ robustness and precision in harsh environments should ensure accurate results. Regular calibration, advanced data analysis, and integration with other monitoring technologies are recommended to enhance performance further. For future research in this field, we recommend the following:

1. Addressing the noise issues in the interrogator resolution is crucial to avoid false alarms in the sensing network and to ensure the reliability of collected data. It is essential to recoat the optical fibre cable with a recoating material that can enhance its adhesion to the surrounding soil, which plays a significant role in the overall performance and longevity of the monitoring system.
2. Future work should focus on developing real-time monitoring and remote sensing by integrating cloud computing and IoT, enhancing sensor performance through advanced algorithms and wireless technologies, and studying soil variability. Testing these systems in large-scale or field models, interdisciplinary collaboration, and field trials will be essential for advancing these technologies.

## 7. Conclusions

The study promoted the integrated science of geotechnical engineering and photonics. A small-scale sinkhole model was designed as a representation of a field sinkhole. A leaking pipe was used to induce the collapse of a cavity, and FBG sensors were placed above the cavity in horizontal and vertical positions to measure the resulting strain variations.

The experimental results suggest that the upward propagation of the sinkhole processes could be divided into the following phases:

Phase 1: Underground cavity formation, determined by the balloon deflation time, produces a stable arch supporting the soil.
Phase 2: Weathering process characterised by the leaking time (water infiltration) after forming the cavity.
Phase 3: Collapsing process, during which time the failure was induced.
Phase 4: Equilibrium period, where the soil mass falls over the sensors and reaches equilibrium.

The FBG sensors are sensitive to ground movement and can identify collapse failure before it becomes visible. Efficient integration of an IoT-enabled FBG sensing system could detect the precursor movement of soil mass due to leaking pipes to guide decision-making and allow for more efficient risk management. The findings also show the viability of OFS for developing an early warning system.

The practical application of OFS technology should be fostered in future monitoring efforts in geohazard-prone areas.

## Figures and Tables

**Table 1:** Scaling factor.

Parameter	Scaling Factor (Prototype/Model)		
		Centrifuge Physical Modelling	1-g Physical Modelling
Displacement, Length		N	N
Mass		N^3^	N^3^
Density		1	1
Stress		1	N
Strain		1	1
Time		N^0.5^	1

**Table 2:** Fundamental engineering properties of silica sand.

Engineering Property	Value
Compaction density	1548.8 kg m^−3^
Relative density	60%
Specific gravity, Gs	2.67
Particle shape	Angular to sub-rounded
Cohesion, C′	0 kPa
Angle of repose (ϕ)	37°
D_50_ particle diameter	0.15 mm
Moisture content	10%
Unified soil classification system (USCS)	SP.

## Data Availability

The original contributions presented in the study are included in the article, further inquiries can be directed to the corresponding author.

## References

[1] Buttrick D.B., Trollip N.Y.G., Gerber A.A. (2011). A Performance Based Approach to Dolomite Risk Management. Environ. Earth Sci..

[2] Gutiérrez F., Parise M., De Waele J., Jourde H., De Waele J., Jourde H., Waele J.D., Jourde H. (2014). A Review on Natural and Human-Induced Geohazards and Impacts in Karst. Earth-Sci. Rev..

[3] Gao Y., Zhu H., Qiao L., Liu X., Wei C., Zhang W. (2023). Feasibility Study on Sinkhole Monitoring with Fiber Optic Strain Sensing Nerves. J. Rock Mech. Geotech. Eng..

[4] Gutiérrez F., Cooper A.H., Johnson K.S. (2008). Identification, Prediction, and Mitigation of Sinkhole Hazards in Evaporite Karst Areas. Environ. Geol..

[5] Zhou W., Beck B.F. (2008). Management and Mitigation of Sinkholes on Karst Lands: An Overview of Practical Applications. Environ. Geol..

[6] Gutiérrez F., Zarroca M., Linares R., Roqué C., Carbonel D., Guerrero J., McCalpin J.P., Comas X., Cooper A.H. (2018). Identifying the Boundaries of Sinkholes and Subsidence Areas via Trenching and Establishing Setback Distances. Eng. Geol..

[7] Parise M. (2015). A Procedure for Evaluating the Susceptibility to Natural and Anthropogenic Sinkholes. Georisk.

[8] Brinkmann R., Wilson K., Elko N., Seale L.D., Florea L., Vacher H.L. (2007). Sinkhole Distribution Based on Pre-Development Mapping in Urbanised Pinellas County, Florida, USA. Geol. Soc. Spec. Publ..

[9] Vaccari A., Stuecheli M., Bruckno B., Hoppe E., Acton S.T. (2013). Detection of Geophysical Features in InSAR Point Cloud Data Sets Using Spatiotemporal Models. Int. J. Remote Sens..

[10] Del Prete S., Di Crescenzo G., Santangelo N., Santo A. (2010). Collapse Sinkholes in Campania (Southern Italy): Predisposing Factors, Genetic Hypothesis and Susceptibility. Z. Geomorphol..

[11] Parise M., Lollino P. (2011). A Preliminary Analysis of Failure Mechanisms in Karst and Man-Made Underground Caves in Southern Italy. Geomorphology.

[12] Intrieri E., Gigli G., Nocentini M., Lombardi L., Mugnai F., Fidolini F., Casagli N. (2015). Sinkhole Monitoring and Early Warning: An Experimental and Successful GB-InSAR Application. Geomorphology.

[13] Doǧan U., Yilmaz M. (2011). Natural and Induced Sinkholes of the Obruk Plateau and Karapidotlessnar-Hotami{dotless}ş Plain, Turkey. J. Asian Earth Sci..

[14] Gao Y., Luo W., Jiang X., Lei M., Dai J. (2013). Investigations of Large Scale Sinkhole Collapses, Laibin, Guangxi, China. Proceedings of the Full Proceedings of the Thirteenth Multidisciplinary Conference on Sinkholes and the Engineering and Environmental Impacts of Karst.

[15] Youssef A.M., Al-Harbi H.M., Gutiérrez F., Zabramwi Y.A., Bulkhi A.B., Zahrani S.A., Bahamil A.M., Zahrani A.J., Otaibi Z.A., El-Haddad B.A. (2015). Natural and Human-Induced Sinkhole Hazards in Saudi Arabia: Distribution, Investigation, Causes and Impacts. Hydrogeol. J..

[16] Baer G., Magen Y., Nof R.N., Raz E., Lyakhovsky V., Shalev E. (2018). InSAR Measurements and Viscoelastic Modeling of Sinkhole Precursory Subsidence: Implications for Sinkhole Formation, Early Warning, and Sediment Properties. J. Geophys. Res. Earth Surf..

[17] Dippenaar M.A., Swart D., Van Rooy J.L. (2019). The Karst Vadose Zone: Influence on Recharge, Vulnerability.

[18] Theron A., Engelbrecht J., Kemp J., Kleynhans W., Turnbull T. (2017). Detection of Sinkhole Precursors Through SAR Interferometry: Radar and Geological Considerations. IEEE Geosci. Remote Sens. Lett..

[19] Constantinou S., Van Rooy J.L. (2018). Sinkhole and Subsidence Size Distribution across Dolomitic Land in Gauteng. J. S. Afr. Inst. Civ. Eng..

[20] Weary D. (2015). The Cost of Karst Subsidence and Sinkhole Collapse in the United States Compared with Other Natural Hazards. Proceedings of the Sinkholes and the Engineering and Environmental Impacts of Karst: Proceedings of the Fourteenth Multidisciplinary Conference.

[21] Gutiérrez F., Guerrero J., Lucha P. (2008). A Genetic Classification of Sinkholes Illustrated from Evaporite Paleokarst Exposures in Spain. Environ. Geol..

[22] Jennings J.E., Brink A.B.A., Louw A., Gowan G. (1965). Sinkholes and Subsidences in the Transvaal Dolomite of South Africa. Int. Soc. Soil Mech. Geotech. Eng..

[23] Jacobsz S.W., Group, T. & F. (2016). Trapdoor Experiments Studying Cavity Propagation. Proceedings of the First Southern African Geotechnical Conference.

[24] Labuschagne J., Ferentinou M., Grobler M., Jacobsz S.W. (2020). Smart Monitoring of Sinkhole Formation Using Optic Fibre Technology. Springer Series in Geomechanics and Geoengineering.

[25] Ferentinou M. (2020). Sinkhole Collapse Propagation Studies through Instrumented Small-Scale Physical Models. Int. Assoc. Hydrol. Sci..

[26] Möller T., da Silva Burke T.S., Xu X., Della Ragione G., Bilotta E., Abadie C.N. (2022). Distributed Fibre Optic Sensing for Sinkhole Early Warning: Experimental Study. Géotechnique.

[27] Terzaghi K. Stress Distribution in Dry Ans in Saturated Sand above a Yielding Trap-Door. https://www.issmge.org/publications/publication/stress-distribution-in-dry-and-in-saturated-sand-above-a-yielding-trap-door.

[28] Costa Y.D., Zornberg J.G., Asce M., Benedito B.S., Costa C.L. (2009). Failure Mechanisms in Sand over a Deep Active Trapdoor. J. Geotech. Geoenviron. Eng..

[29] Alrowaimi M., Yun H.-B., Chopra M. (2015). Sinkhole Physical Models to Simulate and Investigate Sinkhole Collapses. Proceedings of the Sinkholes and the Engineering and Environmental Impacts of Karst: Proceedings of the Fourteenth Multidisciplinary Conference.

[30] Al-Naddaf M., Han J., Jawad S., Abdulrasool G., Xu C. Investigation of Stability of Soil Arching under Surface Loading Using Trapdoor Model Tests. Proceedings of the ICSMGE 2017—19th International Conference on Soil Mechanics and Geotechnical Engineering.

[31] Bado M.F., Casas J.R. (2021). A Review of Recent Distributed Optical Fiber Sensors Applications for Civil Engineering Structural Health Monitoring. Sensors.

[32] Xu D.-S., Dong L.-J., Borana L., Liu H.-B. (2017). Early-Warning System with Quasi-Distributed Fiber Optic Sensor Networks and Cloud Computing for Soil Slopes. IEEE Access.

[33] Li R., Tan Y., Chen Y., Hong L., Zhou Z. (2019). Optical Fiber Technology Investigation of Sensitivity Enhancing and Temperature Compensation for Fi Ber Bragg Grating (FBG)-Based Strain Sensor. Opt. Fiber Technol..

[34] Zomberg R., Hall P., Tech V. (1997). Testing of Reinforced Slopes in a Geotechnical Centrifuge. Geotech. Test. J..

[35] Ni P., Wang S., Zhang S., Mei L. (2016). Response of Heterogeneous Slopes to Increased Surcharge Load. Comput. Geotech..

[36] Park H.J., Kim D.S. (2013). Centrifuge Modelling for Evaluation of Seismic Behaviour of Stone Masonry Structure. Soil Dyn. Earthq. Eng..

[37] Buttrick D.B., Van Schalkwyk A. (1995). The Method of Scenario Supposition for Stability Evaluation of Sites on Dolomitic Land in South Africa, Technical Paper. J. S. Afr. Inst. Civ. Eng..

[38] (2011). Construction Works Part DP1: Earthworks for Buried Pipelines and Prefabricated Culverts; Edition 1.1.

[39] Heath G.J.J., Oosthuizen A.C.C. (2016). A Preliminary Overview of the Sinkhole Record of South Africa. SAICE Conf. Probl. Soils South Afr..

[40] Sverdrup H. (2009). Chemical Weathering of Soil Minerals and the Role of Biological Processes. Fungal Biol. Rev..

[41] Zhang D., Xu Q., Bezuijen A., Zheng G., Wang H. (2017). Internal Deformation Monitoring for Centrifuge Slope Model with Embedded FBG Arrays. Landslides.

